# Investigating the Role of Surface Materials and Three Dimensional Architecture on *In Vitro* Differentiation of Porcine Monocyte-Derived Dendritic Cells

**DOI:** 10.1371/journal.pone.0158503

**Published:** 2016-06-30

**Authors:** Sofie Bruun Hartmann, Soumyaranjan Mohanty, Kerstin Skovgaard, Louise Brogaard, Frederikke Bjergvang Flagstad, Jenny Emnéus, Anders Wolff, Artur Summerfield, Gregers Jungersen

**Affiliations:** 1 National Veterinary Institute, Technical University of Denmark, Frederiksberg C, Denmark; 2 Department of Micro- and Nanotechnology, Technical University of Denmark, Kgs. Lyngby, Denmark; 3 Institute of Virology and Immunology (IVI), Mittelhäusern, Switzerland; INSERM, FRANCE

## Abstract

*In vitro* generation of dendritic-like cells through differentiation of peripheral blood monocytes is typically done using two-dimensional polystyrene culture plates. In the process of optimising cell culture techniques, engineers have developed fluidic micro-devises usually manufactured in materials other than polystyrene and applying three-dimensional structures more similar to the *in vivo* environment. Polydimethylsiloxane (PDMS) is an often used polymer for lab-on-a-chip devices but not much is known about the effect of changing the culture surface material from polystyrene to PDMS. In the present study the differentiation of porcine monocytes to monocyte-derived dendritic cells (moDCs) was investigated using CD172a^pos^ pig blood monocytes stimulated with GM-CSF and IL-4. Monocytes were cultured on surfaces made of two- and three-dimensional polystyrene as well as two- and three-dimensional PDMS and carbonised three-dimensional PDMS. Cells cultured conventionally (on two-dimensional polystyrene) differentiated into moDCs as expected. Interestingly, gene expression of a wide range of cytokines, chemokines, and pattern recognition receptors was influenced by culture surface material and architecture. Distinct clustering of cells, based on similar expression patterns of 46 genes of interest, was seen for cells isolated from two- and three-dimensional polystyrene as well as two- and three-dimensional PDMS. Changing the material from polystyrene to PDMS resulted in cells with expression patterns usually associated with macrophage expression (upregulation of *CD163* and downregulation of *CD1a*, *FLT3*, *LAMP3* and *BATF3*). However, this was purely based on gene expression level, and no functional assays were included in this study which would be necessary in order to classify the cells as being macrophages. When changing to three-dimensional culture the cells became increasingly activated in terms of *IL6*, *IL8*, *IL10* and *CCR5* gene expression. Further stimulation with LPS resulted in a slight increase in the expression of maturation markers (*SLA-DRB1*, *CD86* and *CD40*) as well as cytokines (*IL6*, *IL8*, *IL10* and *IL23A*) but the influence of the surfaces was unchanged. These findings highlights future challenges of combining and comparing data generated from microfluidic cell culture-devices made using alternative materials to data generated using conventional polystyrene plates used by most laboratories today.

## Introduction

Dendritic cells (DCs) are a heterogenic group of antigen presenting cells important for induction of immunological responses. *In vitro* generation of DCs is a technique that has been practiced for decades [1]. It can be difficult to harvest sufficient quantities of DCs for characterization and study, and therefore *in vitro* generated monocyte-derived dendritic cells (moDCs) have been used as an alternative. With this technique peripheral blood mononuclear cells (PBMCs) are harvested, and monocytes isolated and cultured with growth factors, such as granulocyte macrophage colony-stimulating factor (GM-CSF) and interleukin 4 (IL-4) [2]. With the central role of DCs as immune modulators and with the observation that cancer patients have DCs with reduced capacity to initiate efficient Th1 immune responses [3,4], controlling the differentiation of DCs may result in generation of cells able to activate *in vivo* immune responses against antigenic targets that otherwise are not recognized as immunogenic. An example of this is Provenge^®^ (Sipuleucel-T), an FDA registered immunotherapy treatment for prostate cancer [5]. Thus, *in vitro* culture systems for controlled DC maturation, differentiation and immune priming hold promise for activation or deactivation of the immune system against antigenic targets which are inaccessible with current immunization protocols.

In the present study, the pig was used as monocyte donor. The reason this animal was chosen is because of the increasing interest in using porcine models for human diseases. The number of research groups working with the porcine model has been steadily expanding for the last 50 years as judged by the number of publications in PubMed within this area. Advantages using the pig over rodents as animal models for human diseases have been summarised in [6] and includes comparable organ size, large number of cells that can be harvested repeatedly, and blood physiology being similar to humans. The porcine immune system also resembles the human immune system with respect to toll-like receptors (TLR) and DC biology [7]. Porcine DCs have been characterised in several studies [8–14], and much information is already available making the use of this animal as a model for *in vitro* DC differentiation more attractive.

Today most *in vitro* cell studies and cultures are performed using standard flat-bottom polystyrene (PS) culture plates/flasks. This however, constitutes a very non-physiological environment for the cells to grow in and therefore new three dimensional (3D) culture-systems for mammalian cells are gaining increasing interest. Cells cultured in a 3D environment, are largely thought to behave more like *in vivo* compared to cells cultured on a two dimensional (2D) surface, thus making data on gene expression, motility and activity more reliable and comparable to *in vivo* settings (reviewed in [15,16]).

An emerging field within the cell culture area is the use of fabricated micro devices for the study of cells or tissues. By the use of microfluidics, these lab-on-a-chip devices allows for the study of cells using only a small fraction of reagents, and cell analysis can be performed directly in the chip [17]. However, these devises are typically not produced in PS due to difficulties of production, including challenges associated with bonding of thermoplastic materials and production of molds capable of resisting high temperatures and pressures used in hot embossing processes [18]. Alternative polymers with properties suitable for e.g. soft lithography are preferably used. One of these polymers often used is polydimethylsiloxane (PDMS) [19]. Since most reported *in vitro* results on cell activity/behaviour originates from cells cultured on PS surfaces, not much is known about the effect of changing the culture surface material from PS to other materials more frequently used within micro devise fabrication. However, a few studies do exist, and it has previously been reported that PDMS affects the gene expression of adrenal phaeochromocytoma (PC12) cells [20]. The authors found that when cells were cultured on polymethyl methacrylate (PMMA) 6 genes were up-regulated and 35 were down-regulated compared to cells cultured on a PS surface. By contrast, when cells were cultured on a perforated PMMA surface with a PDMS layer underneath 642 genes were up-regulated and 35 were down-regulated compared to cells cultured on PS. They conclude that PDMS opposed to PMMA has a major impact on the gene expression of PC12 cells. Many of the genes differently expressed in the PC12 cells were involved in neuronal cell development and function and so it would be interesting to find out if PDMS had a similar effect on the differentiation of porcine dendritic cells, thus giving rise to cells with a more differentiated and activated phenotype. Therefore, in the present study the differentiation of monocytes to moDCs was investigated using CD172a^pos^ pig blood monocytes stimulated 4 days with GM-CSF and IL-4 followed by 24 hr activation with lipopolysaccharide (LPS). Monocytes were cultured on PS, PDMS and a pyrolysed (carbonised) form of PDMS [21]. Additionally, 2D culture surfaces were compared with 3D scaffolding variants. The purpose of this study was to explore possible changes in gene expression of moDCs when generated on alternative culture surfaces, including materials preferably used for fabrication of micro devices, and 3D vs. 2D architecture.

## Materials and Methods

### 2.1. Isolation of PBMCs

Approximately 100 ml of venous blood drawn from vena jugularis externa by vacupuncture into heparin-coated vacutainer tubes was collected from three 7–8 weeks old pigs (Yorkshire-Landrace x Duroc crossing). The pigs were housed in a common pen with straw bedded concrete floor, ad libitum access to water and fed twice daily with standard pelleted pig feed at the animal facility at The Veterinary Institute, Technical University of Denmark. PBMCs were isolated from the collected whole blood using Ficoll-Paque^™^ Plus (GE Healthcare) and following two washes, cells were counted using an automated cell counter (Nucleocounter^®^ NC-200^™^, ChemoMetec) and diluted in RPMI 1640 medium with GlutaMAX^™^ (Gibco, Life Technologies^™^) containing 10% heat inactivated fetal calf serum (FCS). Animals were kept and sampled under approval by The National Animal Experiments Inspectorate.

### 2.2. Monocyte enrichment

Enrichment of CD172a (SWC3) positive monocytes was done using the MACS magnetic separation technology (Miltenyi Biotec). Briefly, PBMCs were resuspended in MACS buffer (PBS/5xEDTA/1%FCS) and labelled with mouse anti-porcine CD172a IgG1 (clone 74-22-15) at 5.5 μg/10^6^ target cells. After 20 min incubation on ice followed by two washes, MACS magnetic anti-mouse IgG microbeads was added (2 μl/10^6^ target cells) and cells were incubated on ice for 15 min and washed twice. The CD172a positive cells were purified using equilibrated LS columns placed in a magnet on a MACS multistand. The positive fraction was collected, washed, counted and resuspended in complete cell culture medium (see below). The purity was confirmed by incubating with phycoerythrin (PE)-conjugated goat anti-mouse IgG1 secondary antibody (Southern Biotechnology) followed by analysis on a BD FACSCanto^™^ II flow cytometer (BD Biosciences, USA) and was found to be above 94% (data not shown).

### 2.3. Preparation of cell culture surfaces

**Fig 1:**
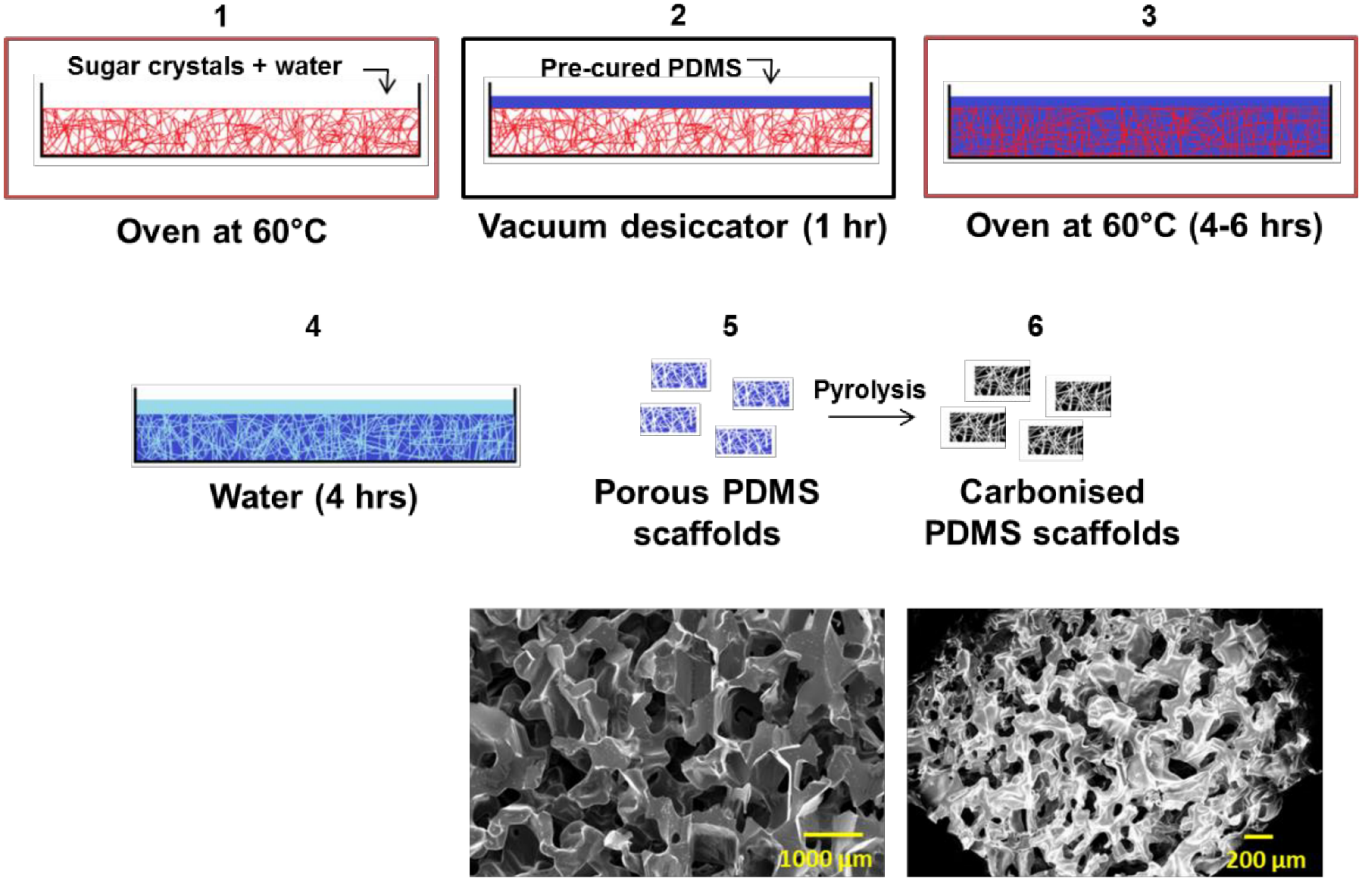
3D PDMS and pyrolysed 3D PDMS scaffold manufacturing. Sugar crystals mixed with water was incubated at 60°C to interconnect the sugar crystals (1), pre-cured PDMS was poured on top of the sugar and placed in a vacuum desiccator (2) to let the PDMS infiltrate the sugar. After an additional incubation at 60°C (3) the PDMS/sugar cube was soaked in water (4) to dissolve the sugar. Disks of random micro-porous PDMS were cut to fit the wells of a 24 well-plate (5). 3D PDMS scaffolds made from square sugar cubes were incubated at 900°C in N_2_ atmosphere resulting in carbonised 3D random micro-porous PDMS (6). Bottom photographs are SEM images showing a 3D PDMS scaffold (left) and pyrolysed 3D PDMS scaffold (right).

The five different surfaces tested in the present experiment are listed in Table 1. Microtiter plates were from Corning Scientific and were either in 6-well (cat# 3516) or 24-well (cat# 3526) format for the 2D and 3D incubations, respectively. They were made in polystyrene, thus empty well on the 6-well plates represented 2D PS surfaces (control). The 3D PS surfaces were made by placing a porous polystyrene 3D scaffold, Alvetex^®^ (Reinnervate) in the bottom of the wells in 24-well plates. For the 2D PDMS surface, PDMS pre-polymer was mixed with a curing agent (10:1, w/w) according to the manufacturer’s instructions (Sylgard^®^ 184, Dow Corning Corporation) and poured in a thin layer into the wells of 6 well plates followed by curing in an oven at 60°C for 4 hours. The random pore 3D PDMS scaffolds were fabricated using particle leaching technique as described previously [22,23] (Fig 1). Briefly, 10 gram of sugar crystals of size ranging from 300–600μm (Sigma-Aldrich) was mixed with 1ml of water on a 100 mm Petri dish. The mixture was placed in an oven at 60°C to generate interconnections between the sugar crystals and to evaporate the excess water. The hardened sugar cube was cooled at room temperature. The PDMS pre-polymer was mixed with a curing agent (10:1, w/w) and poured into the Petri dish containing the sugar cube. The PDMS covered sugar cube was placed in a vacuum desiccator for one hour to let the PDMS infiltrate into the pores of the sugar cube. The PDMS was cured in an oven at 60°C for 4–6 hours. The sugar particles were then dissolved by soaking in water for 4 hours. After dissolving, random micro-porous 3D PDMS scaffolds were cut to cylinders having a diameter of 6 mm and a height of 5 mm, fitting into wells of 24-well plates, using biopsy punches. Both the 2D PDMS surface and the 3D PDMS scaffolds were treated with oxygen plasma (125 W, 13.5 MHz, 50 sccm, and 40 millitorr) to render their surface hydrophilic and sterilised by autoclaving. The pyrolysed 3D PDMS scaffolds were made by pyrolysing the 3D PDMS scaffolds made by square sugar cubes in a furnace at 900°C for one hour in N_2_ atmosphere, resulting in highly porous and hydrophilic square 3D carbonised scaffolds [21]. The morphology and microstructure of PDMS and carbonised PDMS porous scaffolds were examined using scanning electron microscopy (SEM) (JEOL Ltd.). Prior to SEM analysis scaffolds were dried in an oven at 60°C overnight and sputter coated with gold. Samples were then analysed using 12 kV of accelerating voltage. Pore sizes of the moulds and scaffolds were evaluated from SEM micrographs using Image J software.

**Table 1:** Overview of culture surfaces tested.

Surface		Description
2D PS	Two-dimensional polystyrene	6-well plate with no insert (Corning Scientific)
2D PDMS	Two-dimensional polydimethylsiloxane	PDMS moulded in the wells of 6-well plates creating a thin surface layer
3D PS	Three-dimensional polystyrene	Alvetex^®^ discs inserted into wells of 24-well plates (Reinnervate)
3D PDMS	Three-dimensional polydimethylsiloxane	Random micro-porous scaffolds inserted into wells of 24-well plates [22,23]
Pyrolysed 3D PDMS	Pyrolysed three-dimensional polydimethylsiloxane	Carbonised random micro-porous scaffolds inserted into wells of 24-well plates [21]

### 2.4. Cell culture and harvesting

Cells were kept in culture medium (DMEM minus phenol red, high glucose containing 1xNEAA, 1xsodium pyruvate, 1xGlutaMax^™^ supplement, penicillin/streptomycin (100 U/ml and 100 μg/ml, respectively), Fungizone (2μg/ml) (all from Gibco/Life Technologies) and 10% porcine serum (Biochrom)). Prior to cell plating the 3D PS surface was made hydrophilic by adding 70% ethanol followed by two washes with PBS. The 3D PDMS and pyrolysed 3D PDMS surfaces were prepared by washing them twice in PBS. For the 3D surfaces, 1 or 2 x10^6^ cells (for flow cytometry or gene expression analysis, respectively) in a 100–300μl volume was carefully placed on top of the scaffolds, and the plates were pre-incubated at 37°C, 5% CO^2^ for 1 hour in order for the cells to settle within the scaffold. Media containing recombinant porcine GM-CSF and IL-4 (R&D Systems) at a final concentration of 100 and 25ng/ml, respectively, was added to reach a final volume of 2ml per well. Plating of cells on 2D surfaces was done similarly except for the 1 hour pre-incubation step. After three days of incubation, additional 500μl of fresh medium containing GM-CSF and IL-4 was added to all cultures. On day four, half of the cultures received 50μl of LPS (Sigma) at a final concentration of 1μg/ml and were incubated for additional 24 hours. After four days of incubation (five days for the LPS stimulated cultures) cells were harvested by collecting the supernatant and adding ice-cold PBS/5xEDTA to the wells followed by 15 min incubation on ice, shaking. The scaffolds from the 3D cultures were placed in 15 ml tubes (the 3D PS scaffolds were placed in a pipette tip in a 15 ml tube), 1 ml ice-cold PBS/5xEDTA was added and tubes were centrifuged (5 min, 5°C, 500 x g). The cell solution was pooled with the supernatant, centrifuged (5 min, 5°C, 500 x g) and resuspended in RLT buffer (Qiagen) and stored at -80°C for later gene expression analysis or resuspended in FACS buffer (see below) for characterization using flow cytometry. For visualisation of cell morphology, moDCs and monocytes (cultured in media with or without differentiating cytokines, respectively) cultured on 2D PS surfaces were inspected using an inverted Leica DMIL contrasting microscope equipped with a Leica DFC290 camera (Leica Microsystems) at day 1, 4, and 5.

### 2.5. Flow cytometry

For characterization of surface marker expression, cells were stained and analysed by flow cytometry. Cells were distributed in 96-well plates and washed once in FACS buffer (PBS, 0.1% sodium azide, 1% FCS) and incubated 20 min in the dark with 20μl primary antibodies or isotype controls in four antibody cocktails, see Table 2. Following three washes, cells were incubated 20 min in the dark with 20μl secondary antibodies, as listed in Table 2. For compensation adjustments and viability check, pooled cells were stained with either mouse anti-porcine CD172a IgG1 in combination with PE-, APC- or FITC-conjugated anti-mouse IgG1 antibodies, biotin-conjugated CD152 (CTLA-4)-muIg in combination with V450-conjugated streptavidin or the dead cell marker 7-aminoactinomycin D (7-AAD) (Sigma-Aldrich). Cells were washed three times and resuspended in FACS sheath fluid containing 0.5% paraformaldehyde. Cells were loaded on a BD FACSCanto^™^ II flow cytometer (BD Biosciences, USA) and the results were analysed using BD FACSDiva software version 8.0.1.

**Table 2:** Staining overview.

**Primary staining**					
**Cocktail #**	**Primary antibody**	**Conjugate**	**Isotype**	**Clone**	**Company**
1	Mouse anti-porcine CD172a	-	IgG1	74-22-15	DTU Vet^a^
	Mouse anti-porcine SLA class II DR	FITC	IgG2b	2E9/13	AbD Serotec
	CD152(CTLA-4)-muIg (fusion protein)	Biotin	IgG2a	-	Ancell
2	Mouse anti-porcine CD1	-	IgG2a	76-7-4	VMRD
	Mouse anti-porcine CD14	-	IgG1	CAM36A	VMRD
3	Anti-mouse IgG2b (isotype control)	FITC	-	-	AbD Serotec
	Anti-mouse IgG1 (isotype control)	-	-	-	DAKO
	Anti-mouse IgG2a (isotype control)	-	-	-	DAKO
4	muIgFc control (fusion protein)	Biotin	-	-	Ancell
**Secondary staining**					
**Cocktail #**	**Secondary antibody**	**Conjugate**	**Isotype**	**Clone**	**Company**
1	Goat anti-mouse IgG1	PE	-	-	Southern Biotechnology
	Streptavidin	V450	-	-	BD Biosciences
2	Goat anti-mouse IgG2a	FITC	-	-	Southern Biotechnology
	Goat anti-mouse IgG1	APC	-	-	Southern Biotechnology
3	Goat anti-mouse IgG1	APC	-	-	Southern Biotechnology
	Goat anti-mouse IgG1	PE	-	-	Southern Biotechnology
	Goat anti-mouse IgG2a	FITC	-	-	Southern Biotechnology
4	Streptavidin	V450	-	-	BD Biosciences

^a^Produced at The Veterinary Institute, Technical University of Denmark. FITC, Fluorescein isothiocyanate; PE, Phycoerythrin; APC, Allophycocyanin.

### 2.6. RNA extraction

Total RNA was purified from RLT-buffered cell lysates using the RNeasy Mini kit (Qiagen) including an on-column DNase treatment (Qiagen) according to the manufacturer’s instructions. The RNA quantity and quality was measured using a NanoDrop ND-1000 spectrophotometer (Saveen Biotech) and the Agilent 2100 Bioanalyzer (Agilent Technologies), respectively. The average yield of total RNA was (in nanogram ± SD): 2D PS (3698 ± 734); 2D PDMS (5724 ± 785); 3D PS (275 ± 273); 3D PDMS (1340 ± 238) and pyrolysed 3D PDMS (1096 ± 381). The RNA integrity number (RIN) was determined using the Agilent RNA 6000 Nano Kit (Agilent Technologies) according to the manufacturer’s instructions. All RIN numbers were between 8.1 and 10.

### 2.7. cDNA synthesis and pre-amplification

QuantiTect^®^ reverse Transcription Kit (Qiagen) was used to reverse transcribe 180ng total RNA in duplicate according to the manufacturer’s instructions. For the pre-amplification, cDNA was diluted 1:5 in low EDTA Tris-EDTA (TE)-buffer (VWR-Bie & Berntsen). TagMan PreAmp Master mix (1μl) (Applied Biosystems) was mixed with 2.5μl of a 200nM primer mix (containing all primer pairs used in this study) and 2.5μl diluted cDNA. Using a Thermocycler (Biometra), samples were incubated at 95°C for 10 min followed by 18 cycles of 95°C for 15 sec and 60°C for 4 min. For removal of excess primers, exonuclease I (New England Biolabs) (4U/μl) was added to the pre-amplified cDNA and samples were incubated at 37°C for 30 min and 80°C for 15 min. Pre-amplified, exonuclease-treated cDNA was diluted 1:5 in low EDTA TE-buffer and stored at -20°C until quantitative real time PCR (RT-qPCR) analysis.

### 2.8. Primer design and validation

Primers targeting genes involved in differentiation and activation were designed using the free software Primer3 (http://bioinfo.ut.ee/primer3-0.4.0/primer3/) as described previously [24] and synthesized at TAG Copenhagen (Copenhagen, Denmark) or Sigma-Aldrich (Denmark). All primers, primer sequences and amplicon length are listed in S1 Table. If possible, primers were designed to span introns to prevent amplification of genomic DNA, and BLAST searches were performed to ensure primer specificity and absence of intraspecies polymorphisms at the primer site. For several genes, two primer pairs, annealing at different sites at the mRNA transcript, were designed. Primer amplification efficiencies and correlation coefficients were obtained by standard curves constructed from four separate dilution series of pooled pre-amplified, exonucleated cDNA. To ensure primer specificity, melting curves were visually inspected for all primer assays.

### 2.9 Quantitative real-time PCR

qRT-PCR was performed in 96.96 Dynamic Array Integrated Fluidic Circuits (IFC) (Fluidigm), combining 54 samples, 38 dilution curve samples, three minus reverse transcriptase controls and one non-template control, all pre-amplified (96 samples in total) with 96 primer sets for 9216 individual and simultaneous qPCR reactions. Reaction mix was prepared using the following reagents for each of the 96 reactions: 3μl TaqMan Gene Expression Master Mix (Applied Biosystems), 0.3μl 20XDNA Binding Dye Sample Loading Reagent (Fluidigm), 0.3μl 20X EvaGreen (Biotium, VWR-Bie & Berntsen) and 0.9μl low EDTA TE buffer. Reaction mix (4.5μl) was mixed with 1.5μl pre-amplified, diluted cDNA. Primer mix was prepared for each of the 96 primer sets using 3μl of 20μM primer (forward and reverse) and 3μl of 2X Assay loading Reagent (Fluidigm). The 96.96 Dynamic Array was primed in the IFC Controller (Fluidigm), and reaction mix including cDNA (5μl) and primer mix (5μl) was loaded into appropriate inlets on the chip, and again placed in the IFC controller to ensure equal distribution of cDNA and primer in the 9216 reaction chambers. The chip was then placed in the BioMark real time PCR instrument (Fuidigm), and the following cycle conditions were used: a thermal mix consisting of 2 min at 50°C, 30 min at 70°C and 10 min at 25°C, then an uracil-N-glycosylase (UNG) phase with 2 min at 50 and a hot start phase with 10 min at 95°, followed by 35 cycles with denaturation for 15 sec at 95° and annealing/elongation for 1 min at 60°C. Melting curves were generated after each run to confirm a single PCR product (from 60°C to 95°C, increasing 1°C/3 sec). Reactions were performed in duplicate (cDNA replicates). Minus RT controls (samples generated without reverse transcriptase enzyme) and non-template controls (NTC) were included to check for genomic DNA contamination and non-specific amplification, respectively. Expression data (Cq values) was acquired using the Fluidigm Real Time PCR Analysis software 3.0.2 (Fluidigm).

### 2.10. Data pre-processing and analysis

Data pre-processing, normalization and relative quantification was done using GenEx5 (MultiD). Data were corrected for primer efficiency for each individual assay. If primer efficiency was below 0.85 or above 1.15, a default efficiency of 0.90 was assigned and differential expression was later validated using the Rotor-Gene Q (Qiagen) qPCR platform. Normalization was done using the geometric mean of four reference genes: *hypoxanthine phosphoribosyl-transferase 1 (HPRT1)*, *tyrosine 3-monooxygenase/tryptophan 5-monooxygenase (YWHAE)*, *β-actin (ACTB)* and *β-2-microglobulin (B2M*), found to be the most stably expressed ones out of eight reference genes tested using both GeNorm [25] and NormFinder [26]. Data from the two technical cDNA replicates were averaged, and for each specific primer set and sample, a maximum of 10% samples/primer sets with a ΔCq above ±1.5 for the two cDNA replicates was accepted. To visualize differential gene expression, expression was calculated relative to the sample with the lowest expression (highest Cq) within each primer assay. Finally the data was log_2_ transformed and autoscaled before principal component analysis (PCA). No statistical analyses were attempted since only three pigs were analysed in the present study. For comparison of the effect of the different culture surfaces the expression was set relative to the control surface (2D PS) and a fold change of ±2 was arbitrarily defined as the cut-off for biologically significant changes.

## Results

### 3.1. Monocytes cultured conventionally with differentiating cytokines have moDC morphology and surface marker expression

**Fig 2:**
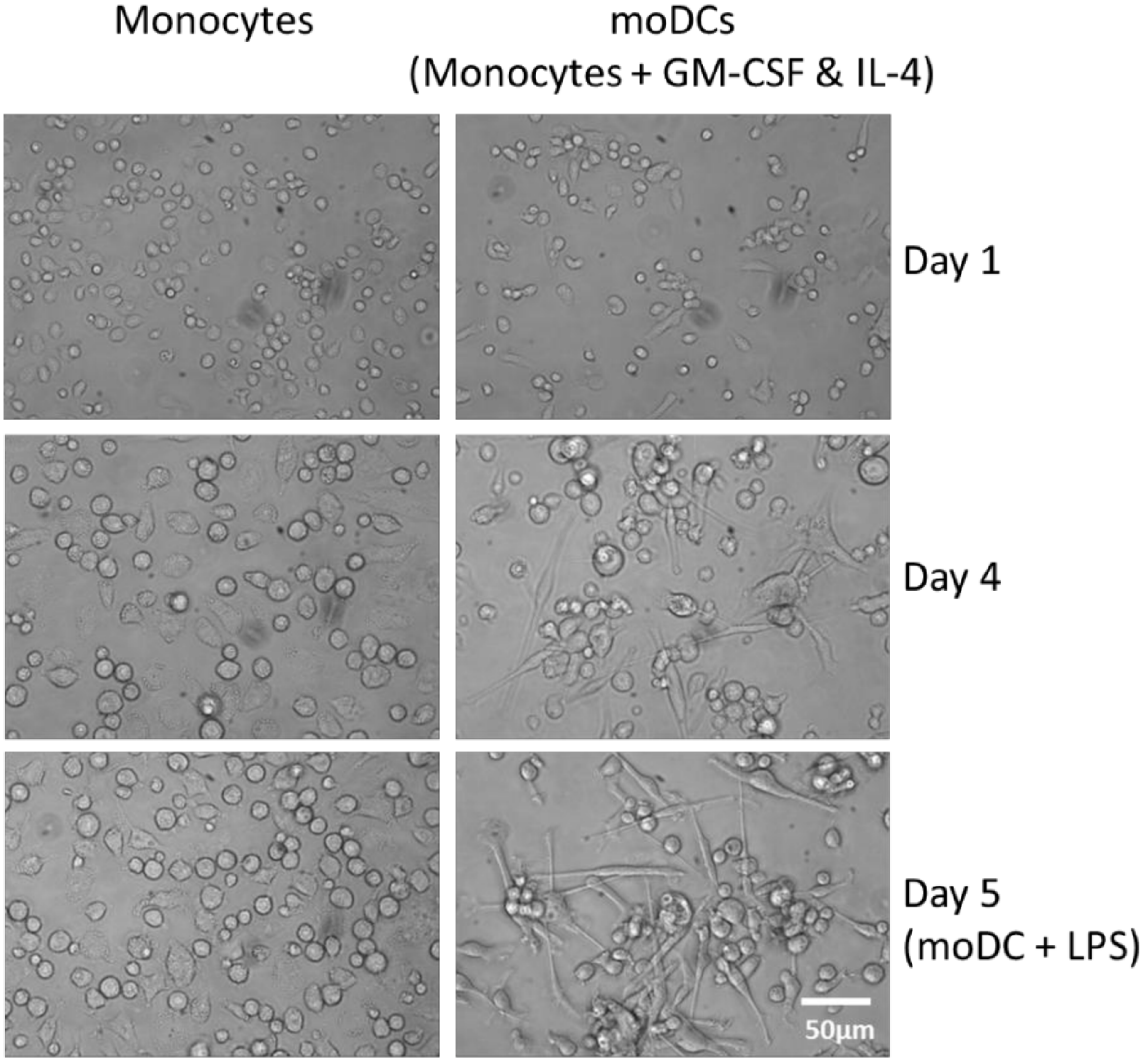
Light microscopy images of monocytes and moDCs after 1, 4 and 5 days of incubation on a 2D PS surface. At day 5 the moDCs had been stimulated with LPS for 24 hours. MoDCs were generated by addition of differentiating cytokines GM-CSF and IL-4. MoDC, monocyte-derived dendritic cells; LPS, lipopolysaccharide; GM-CSF, Granulocyte-macrophage colony-stimulating factor; IL-4, interleukin 4.

**Fig 3:**
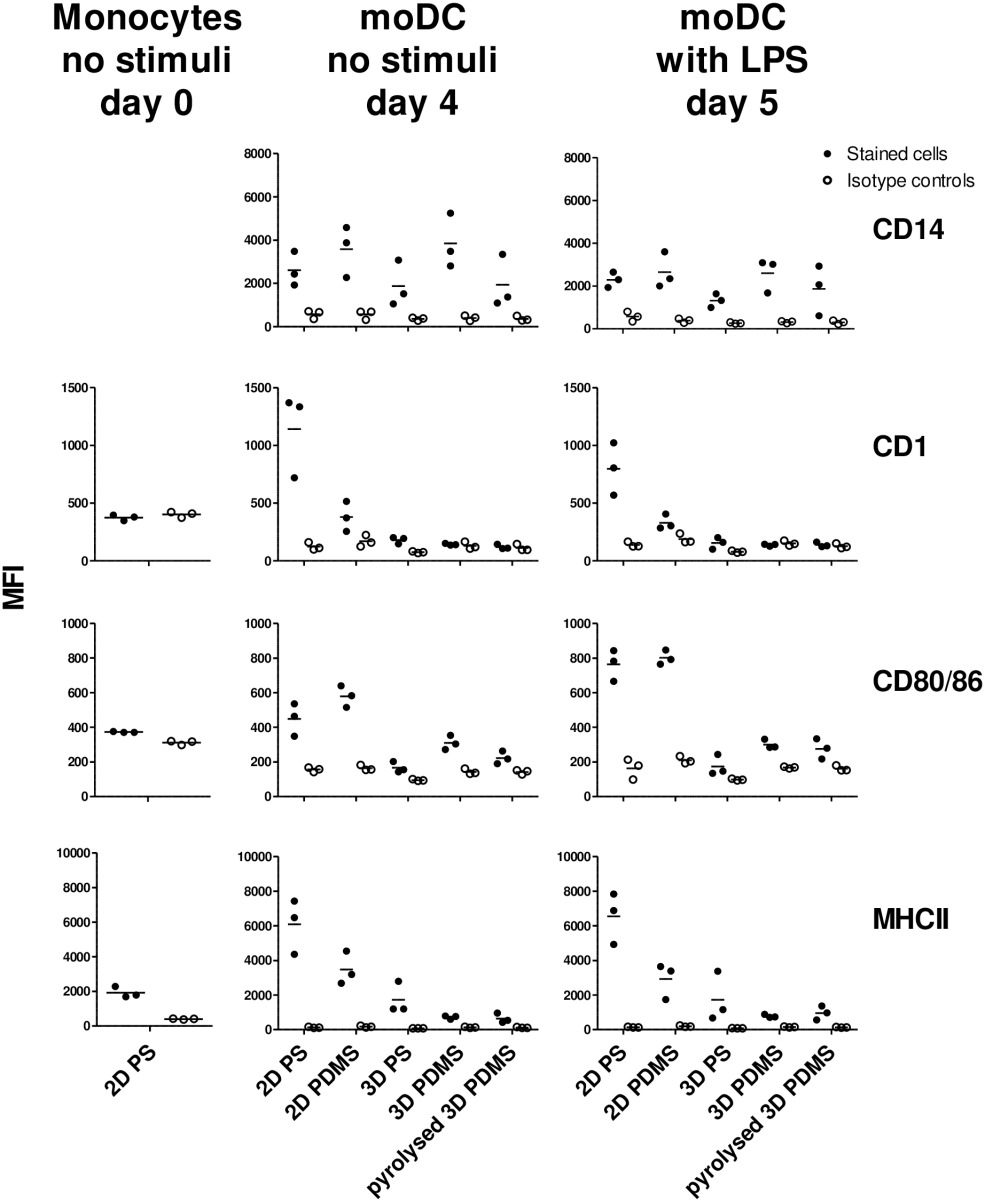
Surface marker expression of porcine monocytes compared to non-stimulated (day 4) and LPS-stimulated (day 5) moDCs cultured on different surfaces. Shown is the median fluorescence intensity (MFI) of individual surface markers (n = 3). Filled circles represent relevant antibody-stained samples, open circles represents isotype controls. It was not possible to detect CD14 on monocytes as this antibody had same isotype (IgG1) as the antibody used to purify the monocytes thus giving rise to false positives. PS, polystyrene; PDMS, polydimethylsiloxane; LPS, lipopolysaccharide.

In the present study, the effect of culture surface architecture and material on the *in vitro* differentiation of porcine moDC was investigated. For this purpose three pigs were used as donors. Purified CD172a positive monocytes were cultured with medium containing GM-CSF and IL-4 on surfaces that differed in material; PS, PDMS or carbonised (pyrolysed) PDMS, and dimension; 2D or 3D. After four days of culture on 2D PS in the presence of differentiating cytokines, monocytes differentiated into loosely adhenrent, elongated cells with dendrites—a morphology characteristic of moDC (Fig 2). This was even more profound after 24 hours of activation with LPS. To confirm the differentiation into moDCs the expression of surface markers was analysed by flow cytometry. Prior to culture, monocytes were CD1 negative, CD80/86^negative/low^ and MHCII^low^ (Fig 3). After conventional (2D PS) culture with differentiating cytokines, cells were CD1^pos^, CD80/86^high^ (especially after activation by LPS) and MHCII^high^ which further confirmed their differentiation into moDCs [9,11,12]. When cultured on 3D surfaces of both materials however, cells were CD1^neg^, CD80/86^low^ and MHCII^low^, a phenotype resembling monocytes more than moDCs. Cells cultured on 2D PDMS had an intermediate phenotype; CD1^low^, CD80/86^high^ and MHCII^high^. CD14 was equally expressed by cells cultured on all five surfaces.

### 3.2. Gene expression

The expression of 96 genes—88 genes of interest and 8 reference genes was examined. Genes were selected to cover transcription factors, cytokines, maturation markers and phenotypic markers. The following genes were excluded due to expression levels below limits of detections or variable cDNA replicates: *IL12(p35)*, *IL12(p40)*, *IFN-β*, *IFN-γ*, *ITGAM(a)*, *ITGAM(b)*, *CXCL10*, *IL1B*, *TNF*, *CLEC4A(b)*, *SIGLEC5(a)*, *CCL2*, *ITGA4(b)*, *CLEC1A(b)*, *CLEC12A(a)*, *CLEC12A(b) and CD101(b)*. Validation of differential expression was performed on 11 primers assigned with default efficiency. Expression levels from primers specific for *TLR3* could not be validated on the Rotor-Gene Q qPCR platform and was thus excluded from the analysis. For genes analysed using two primer pairs, only one assay was analysed further after checking the correlation coefficients between the two pairs. Thus, the following genes were excluded due to double primer assay (correlation coefficient between the two primer sets are shown in parentheses): *TLR4(a)* (0.81), *SLA-DRB1(b)* (0.95), *CD86(b)* (0.94), *CCR7(b)* (0.98), *TRAF6(a)* (0.97), *CCR5(b)* (0.97), *CXCR4(b)* (0.83), *IRF8(b)* (0.92), *FLT3(a)* (0.99), *CCR1(a)* (0.88), *IRF5(a)* (0.65), *BATF3(a)* (0.65), *ID2(a)* (0.97), *BCL11A(a)* (0.77), *BCL6(a)* (0.97), *TCF4(b)* (0.86), *CLEC2D(b)* (0.88), *CD163(b)* (0.99), *FCGR1A(a)* (0.82), *FCGR2B(a)* (0.96), *FCGR3B(a)* (0.83), *CD1a(b)* (0.99), *LAMP3(b)* (0.91) and *XCR1(a)* (0.91). Thus, a total of 46 genes were evaluated for differential gene expression (many of those with two highly correlated primer pairs targeting the same mRNA transcript) as a result of surface architecture (2D vs. 3D) and materials used for culturing. (see suppl. S1 Table for complete list of primers).

### 3.3. The monocyte-derived cells show distinct gene expression when cultured on surfaces of alternative materials and dimension.

**Fig 4:**
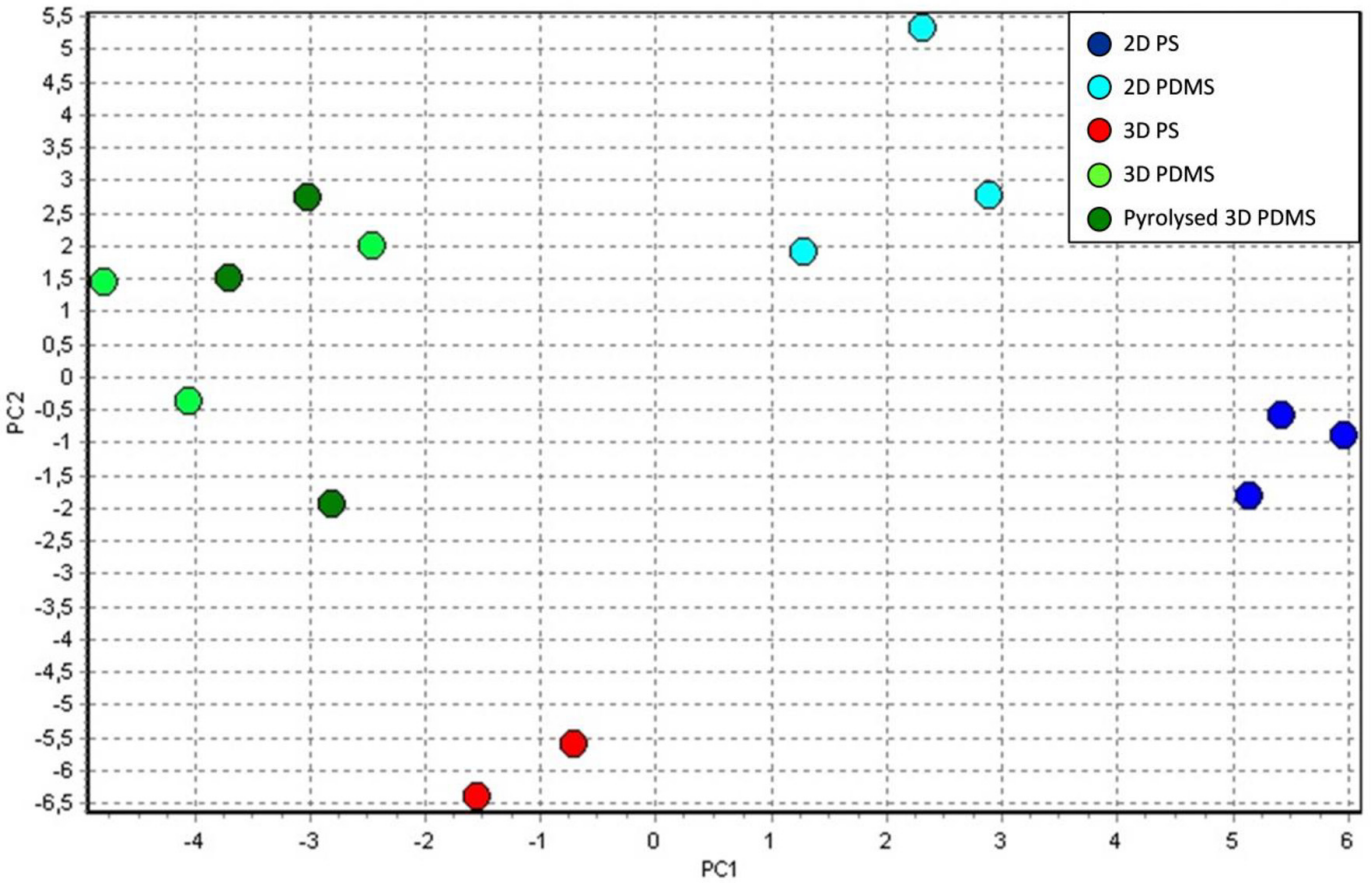
Principal component analysis on LOG2, autoscaled expression data of all 46 genes. Distinct clustering of cells isolated from 2D PS (dark blue), 2D PDMS (light blue), 3D PS (red) and 3D PDMS/pyrolysed 3D PDMS (green) can be seen. The analysis is based on data from 3 pigs, except for the 3D PS culture where 2 pigs were analysed. Non-stimulated cells were used for the analysis.

**Fig 5:**
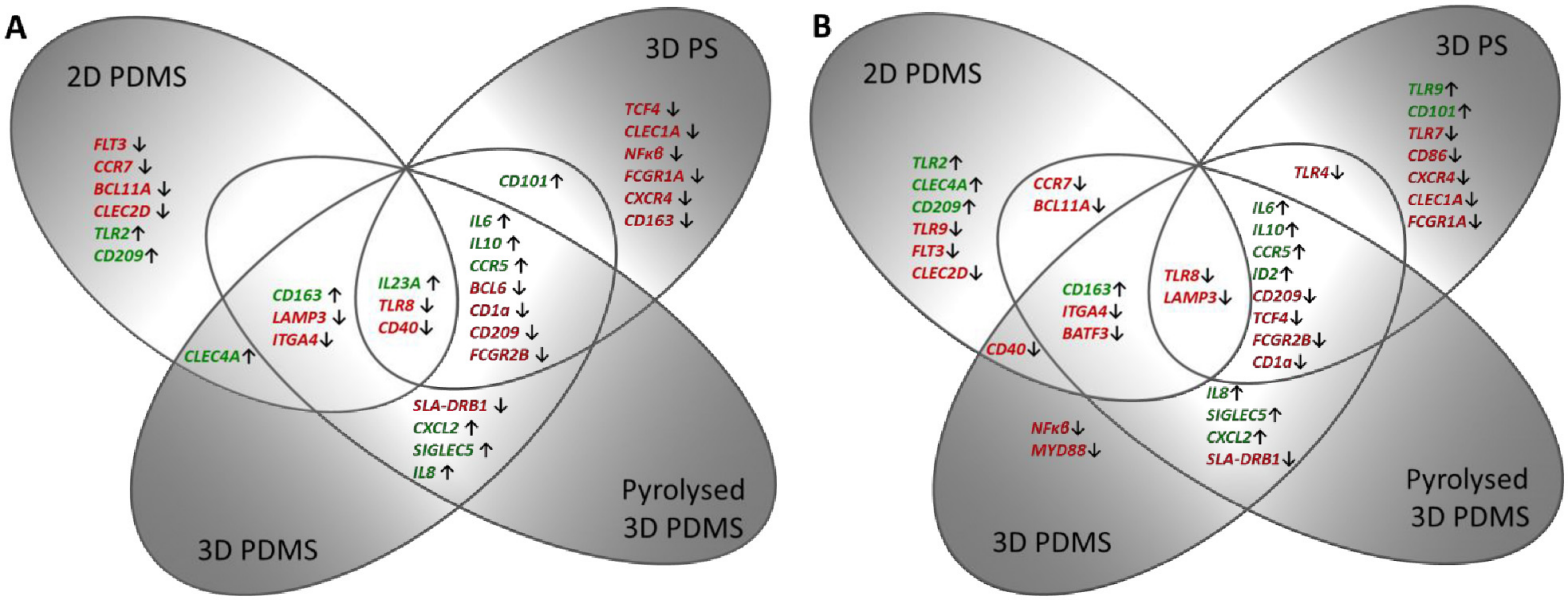
Schematic illustration of the effect of culture surface on the gene expression of moDCs. Genes with at least a two-fold up- or down-regulation (see Table 3) on the four alternative surfaces compared to conventional surface (2D PS) are shown for non-stimulated cells (A) and LPS-stimulated cells (B). Red = down-regulated genes, green = up-regulated genes.

Principal component analysis (PCA) was performed on relative expression data of all 46 genes, to reveal patterns or clusters among samples based on co-expression patterns. Distinct clustering of cells isolated from 2D PS, 2D PDMS, 3D PS and 3D PDMS/pyrolysed 3D PDMS can be seen in Fig 4. Most variation was explained by PC1 (eigenvalue = 30.4%) separating the cells grown on 2D compared to 3D. No separation, based on gene expression patterns, was seen between cells grown on the carbonised form of 3D PDMS and 3D PDMS. To see how the gene expression changed when cells were cultured on alternative surfaces, the expression was compared relative to the expression on the conventional control surface (2D PS), set to 1. Both the gene expression from non-stimulated- and LPS-stimulated cells is shown in Table 3. Out of the 46 genes analysed, 29 genes had a fold change expression of ± 2 when cultured alternatively compared to control, and for the LPS-stimulated cells this number was 34. In agreement with results from the PCA, the two most variable groups of clusters (2D PS and 3D PDMS) also showed the highest number of differentially expressed genes both in samples of no stimuli and LPS treatment. Fig 5 summarises the differently expressed genes across the alternative surfaces, with or without LPS. In the non-stimulated cells, a common effect of changing the material from PS to PDMS (both 2D-, 3D- and pyrolysed 3D PDMS) was increased expression of *CD163* and decreased expression of *LAMP3* (*CD208*) and *ITGA4* (*CD49d*). The same applied for the LPS-stimulated cells, except here also the expression of *BATF3* was decreased in cells from the PDMS surfaces and the expression of *LAMP3* was, in addition to PDMS surfaces, also decreased in cells cultured on 3D PS. A common effect of changing the dimension from 2D to 3D in resting cells (both PS, PDMS and pyrolysed PDMS) was increased expression of *IL6*, *IL10*, *CCR5* and decreased expression of *BCL6*, *CD1a*, *CD209* (*DC-SIGN*) and *FCGR2B* (*CD32*). In the LPS-stimulated cells this was also the case only expression of *BCL6* was unchanged compared to control, and in addition the expression of *ID2* and *TCF4* was increased and decreased, respectively. The expression of *SLA-DRB1* (*MHCII*) was approximately the same in cells from 2D PDMS and 3D PS, but was decreased 2.3 fold (0.43) in cells from 3D PDMS and pyrolysed 3D PDMS. The same trend applied for cells stimulated with LPS. The expression of *CD86* was unchanged across the various surfaces, except for a 2 fold (0.46) downregulation in LPS-stimulated 3D PS cells. Common for all the four alternative surfaces tested was an increased expression of *IL23A* and a decreased expression of *TLR8* and *CD40* when compared to the conventional 2D PS culture surface. For the LPS-stimulated cells a decrease in the expression of *TLR8* and *LAMP3* was observed for all four alternative surfaces. The most important findings regarding the gene expression results for all tested surfaces are summarised in Table 4.

**Table 3:** Relative gene expression (fold change) in moDCs from alternative surfaces compared to conventional surface.

	No stimuli (day 4)					LPS (day 5)				
	Control surface	Alternative surfaces				Control surface	Alternative surfaces			
	2D PS	2D PDMS	3D PS^a^	3D PDMS	Pyrolysed 3D PDMS	2D PS	2D PDMS	3D PS^b^	3D PDMS	Pyrolysed 3D PDMS
*IL6*	1±0.21	0.73±0.13	**4.16±1.03**	**10.31±0.99**	**11.87±0.87**	1±0.13	0.92±0.24	**6.03**	**12.12±2.03**	**22.37±4.50**
*IL8*	1±0.12	1.34±0.43	0.71±0.29	**4.25±1.41**	**4.42±1.19**	1±0.24	0.67±0.11	1.31	**2.61±0.42**	**4.02±0.69**
*IL10*	1±0.12	1.90±0.32	**8.43±2.06**	**13.56±2.87**	**18.58±3.56**	1±0.22	1.19±0.12	**7.76**	**9.68±0.88**	**12.63±0.49**
*IL23A*	1±0.16	**3.36±0.60**	**5.53±1.52**	**17.52±2.37**	**10.16±1.73**	1±0.15	0.61±0.07	1.81	1.16±0.38	1.60±0.48
*TLR2*	1±0.20	**2.20±0.22**	0.75±0.24	1.82±0.46	1.77±0.49	1±0.18	**2.35±0.25**	0.82	1.69±0.21	1.91±0.34
*TLR4(b)*	1±0.17	1.40±0.27	0.51±0.08	0.59±0.04	0.58±0.12	1±0.22	1.15±0.14	**0.39**	**0.45±0.07**	0.55±0.07
*TLR7*	1±0.30	1.00±0.31	0.77±0.04	0.91±0.32	0.84±0.26	1±0.32	0.72±0.24	**0.27**	0.71±0.25	0.62±0.17
*TLR8*	1±0.27	**0.09±0.05**	**0.08±0.02**	**0.01±0.00**	**0.03±0.01**	1±0.47	**0.03±0.00**	**0.04**	**0.02±0.01**	**0.01±0.00**
*TLR9*	1±0.53	0.83±0.47	1.34±0.69	1.17±0.60	1.00±0.40	1±0.48	**0.45±0.27**	**2.10**	0.76±0.39	0.87±0.44
*IFNA*	1±0.37	0.99±0.18	1.13±0.26	0.68±0.04	0.94±0.11	1±0.24	0.90±0.12	1.11	0.81±0.16	0.61±0.17
*SLA-DRB1(a)*	1±0.15	0.67±0.14	0.98±0.18	**0.43±0.10**	**0.43±0.11**	1±0.15	0.59±0.13	1.01	**0.32±0.05**	**0.35±0.02**
*CD86(a)*	1±0.16	0.97±0.04	0.67±0.02	0.90±0.06	0.95±0.15	1±0.11	0.76±0.09	**0.46**	0.67±0.04	0.71±0.09
*CD40*	1±0.14	**0.31±0.04**	**0.22±0.01**	**0.50±0.10**	**0.44±0.14**	1±0.17	**0.42±0.04**	0.53	**0.46±0.07**	0.57±0.06
*CCR7(a)*	1±0.40	**0.19±0.07**	1.16±0.60	0.74±0.23	0.57±0.17	1±0.30	**0.13±0.02**	0.61	0.66±0.16	**0.45±0.08**
*TRAF6(b)*	1±0.05	0.83±0.05	0.83±0.10	0.85±0.01	0.83±0.04	1±0.11	0.68±0.08	0.74	0.62±0.04	0.69±0.03
*CLEC4A(a)*	1±0.19	**2.21±0.62**	0.82±0.06	**2.19±0.35**	1.40±0.22	1±0.29	**3.29±0.90**	0.62	1.39±0.36	1.11±0.41
*IRF1*	1±0.08	1.28±0.07	0.78±0.05	1.29±0.23	1.38±0.17	1 ±0.11	0.98±0.04	0.77	0.76±0.04	0.99±0.03
*CCR5(a)*	1±0.23	1.91±0.59	**4.10±0.78**	**8.80±0.33**	**9.18±1.46**	1 ±0.13	1.63±0.35	**8.39**	**9.42±0.63**	**7.38±0.75**
*CXCR4(a)*	1±0.17	1.46±0.28	**0.27±0.01**	1.25±0.27	0.94±0.18	1±0.20	1.79±0.09	**0.41**	1.50±0.13	1.44±0.19
*IRF8(a)*	1±0.02	1.61±0.36	1.07±0.76	1.78±0.65	1.60±0.48	1±0.09	1.24±0.06	0.68	1.58±0.36	1.26±0.44
*FLT3(b)*	1±0.24	**0.40±0.11**	1.85±0.54	1.94±0.46	1.42±0.47	1±0.16	**0.20±0.08**	1.86	1.24±0.30	1.32±0.28
*SIGLEC5(b)*	1±0.07	1.24±0.20	1.54±0.50	**3.04±1.14**	**2.70±0.84**	1±0.28	1.24±0.31	1.49	**2.58±0.57**	**2.60±0.41**
*CD209*	1±0.10	**2.49±0.27**	**0.08±0.01**	**0.18±0.07**	**0.21±0.06**	1±0.18	**2.86±0.40**	**0.03**	**0.14±0.04**	**0.40±0.11**
*ITGA4(a)*	1±0.19	**0.32±0.03**	0.62±0.05	**0.39±0.04**	**0.49±0.05**	1±0.14	**0.23±0.05**	0.54	**0.36±0.05**	**0.49±0.08**
*CCR1(b)*	1±0.08	1.29±0.10	0.87±0.03	1.07±0.10	1.25±0.17	1±0.04	1.39±0.08	0.90	1.17±0.08	1.18±0.12
*IRF5(b)*	1±0.04	1.00±0.21	1.49±0.15	1.02±0.09	1.11±0.20	1±0.11	0.74±0.07	1.45	0.74±0.04	0.88±0.07
*NFKB*	1±0.06	0.59±0.04	**0.44±0.01**	0.59±0.04	0.58±0.03	1±0.06	0.56±0.10	0.59	**0.50±0.07**	0.61±0.08
*BATF3(b)*	1±0.14	0.85±0.05	0.56±0.07	0.83±0.15	0.76±0.14	1±0.20	**0.35±0.15**	0.80	**0.44±0.20**	**0.45±0.19**
*ID2(b)*	1±0.11	1.58±0.18	1.73±0.27	1.71±0.28	1.88±0.24	1±0.11	1.57±0.01	**3.19**	**2.18±0.21**	**2.76±0.07**
*BCL11A(b)*	1±0.15	**0.48±0.08**	0.69±0.14	1.23±0.18	0.82±0.19	1±0.17	**0.39±0.17**	0.86	0.65±0.14	**0.50±0.10**
*BCL6(b)*	1±0.11	0.59±0.06	**0.43±0.08**	**0.47±0.04**	**0.44±0.04**	1±0.18	0.63±0.07	0.57	0.57±0.12	0.64±0.09
*TCF4(a)*	1±0.21	0.73±0.21	**0.45±0.13**	0.51±0.16	0.54±0.13	1±0.24	0.60±0.14	**0.34**	**0.32±0.08**	**0.39±0.09**
*MYD88*	1±0.08	0.73±0.15	0.54±0.06	0.51±0.06	0.66±0.02	1±0.10	0.80±0.07	0.54	**0.43±0.04**	0.53±0.03
*LY96*	1±0.06	1.09±0.16	0.65±0.11	0.97±0.11	0.88±0.06	1±0.04	0.77±0.11	0.73	0.81±0.08	1.03±0.14
*CLEC1A(a)*	1±0.31	1.33±0.59	**0.44±0.04**	0.63±0.17	0.61±0.24	1±0.26	1.67±0.45	**0.41**	0.95±0.07	0.79±0.37
*CLEC2D(a)*	1±0.25	**0.33±0.00**	0.60±0.25	0.84±0.08	0.94±0.17	1±0.10	**0.32±0.02**	0.70	0.51±0.03	0.56±0.04
*CXCL2*	1±0.09	1.68±0.51	1.23±0.48	**5.01±1.42**	**4.82±1.41**	1±0.03	0.62±0.10	1.60	**2.31±0.56**	**3.35±0.95**
*CD163(a)*	1±0.30	**8.74±2.93**	**0.22±0.18**	**6.17±1.61**	**4.70±0.89**	1±0.30	**6.62±1.61**	1.20	**10.07±3.27**	**8.84±1.38**
*IRF3*	1±0.04	1.06±0.05	1.17±0.13	0.98±0.13	0.90±0.05	1±0.22	0.63±0.08	0.96	0.59±0.04	0.74±0.08
*FCGR1A(b)*	1±0.27	1.62±0.89	**0.38±0.10**	0.72±0.30	0.85±0.22	1±0.18	0.87±0.25	**0.30**	0.67±0.17	0.91±0.22
*FCGR2B(b)*	1±0.12	1.19±0.33	**0.17±0.01**	**0.40±0.09**	**0.38±0.11**	1±0.15	1.20±0.12	**0.25**	**0.39±0.09**	**0.42±0.07**
*FCGR3B(b)*	1±0.01	1.84±0.25	0.74±0.08	1.59±0.26	1.43±0.15	1±0.01	1.29±0.01	0.86	1.43±0.14	1.58±0.15
*CD1a(a)*	1±0.13	0.71±0.21	**0.28±0.14**	**0.03±0.00**	**0.05±0.01**	1±0.29	0.58±0.35	**0.06**	**0.02±0.01**	**0.02±0.00**
*LAMP3(a)*	1±0.12	**0.20±0.05**	0.62±0.20	**0.38±0.13**	**0.27±0.07**	1±0.16	**0.08±0.04**	**0.39**	**0.17±0.06**	**0.21±0.07**
*XCR1(b)*	1±0.20	1.12±0.28	0.56±0.11	0.79±0.13	0.81±0.10	1±0.03	1.01±0.12	1.28	0.95±0.06	1.12±0.01
*CD101(a)*	1±0.51	1.88±0.10	**2.05±0.35**	**2.86±0.96**	1.98±1.14	1±0.26	1.15±0.25	**2.65**	1.26±0.36	1.20±0.13
**Genes with ≥2-fold regulation**	**-**	**13**	**17**	**19**	**17**	**-**	**14**	**18**	**21**	**19**

Relative gene expression of moDCs from three pigs cultured on alternative material/scaffold relative to conventional culture surface (= 1), analysed by qPCR±SEM. A two-fold or more regulation is marked by bold case. 2D, two-dimensional; 3D, three-dimensional; PS, polystyrene; PDMS, polydimethylsiloxane; qPCR, quantitative PCR; SEM, standard error of the mean.

^a^Data generated from only two.

^b^Data generated from only one pig.

Please note that the data resulting from no stimuli and LPS treatment cannot be directly compared.

**Table 4:** Summary of findings.

Culture setup	Regulation of selected genes compared to control surface^a^	Activation status of non-stimulated / LPS-stimulated cells
D PS (control)	NA	**- / +**
2D PDMS	*FLT3*^b^↓, *LAMP3*^b^↓, *BATF3*↓^b^^,^^e^, *CD163*^c^↑, *IL23A*↑^d^	**+ / +**
3D PS	*CD1a*^b^↓, *FCGR2B*^b^↓, *CD163*^c^↓, *IL6*^d^↑, *IL10*^d^↑, *IL23A*^d^↑, CCR5^d^↑	**+ + / + + +**
3D PDMS	*LAMP3*^b^↓, *CD1a*^b^↓, *FCGR2B*^b^↓, *BATF3*↓^b^^,^^e^, *CD163*^c^↑, *IL6*^d^↑, *IL8*^d^↑, *IL10*^d^↑, *IL23A*^d^↑, CCR5^d^↑	**+ + / + + +**
Pyrolysed 3D PDMS	*LAMP3*^b^↓, *CD1a*^b^↓, *FCGR2B*^b^↓, *BATF3*↓^b^^,^^e^, *CD163*^c^↑, *IL6*^d^↑, *IL8*^d^↑, *IL10*^d^↑, *IL23A*^d^↑, CCR5^d^↑	**+ + / + + +**

NA, not applicable.

^a^In non-stimulated cells;

^b^DC marker;

^c^macrophage marker;

^d^activation marker;

^e^In LPS-stimulated cells.

## Discussion

This study aimed at investigating the effect of changing the culture conditions when generating porcine moDCs *in vitro*. For this purpose conventional PS culture plates (2D PS) were compared with plates coated with a thin layer of PDMS (2D PDMS). The reason PDMS was chosen was because of its common usage within microfluidic cell culture devices, a field that have undergone significant growth in recent years [17,27,28]. With PDMS it is easy to create highly complex fluidic features [29], a characteristic not shared by polystyrene mainly because of challenges with bonding of the material [30]. The use of plastic ware for bioanalysis has evolved in two directions during the last 20 years; while engineers have become accustomed to use PDMS for fabrication of micro devices, biologists have almost entirely used PS as material of choice for culture ware, e.g. Petri dishes, culture flasks and microtiter plates [18]. Therefore most of the *in vitro* biological data available today is from cells cultured on PS and thus comparison of data generated using other materials might be difficult. There is no doubt that fabricated micro devices for testing biological samples will become increasingly implemented within biomedical research, and thus generating knowledge of how cells respond to growth in/or contact with alternative materials and surface architecture is crucial.

The porcine monocytes-derived cells generated with a cocktail of GM-CSF and IL-4 had a surface phenotype typically for moDC when cultured on 2D PS [9,11,12]. The phenotype when cultured on 2D PDMS was similar to 2D PS-cultured cells except for decreased expression of CD1 and MHCII. All the 3D-cultured cells had no or low expression of CD1, CD80/86 and MHCII. This was consistent with the gene expression analysis; when compared to control, the expression of *CD1a* was slightly decreased in 2D PDMS cells (a fold change of 1.4 (a relative-to-control expression of 0.71) which was, however, not categorized as being biologically significant; see material and methods), and decreased 3.5–33 fold (0.28–0.03) in cells from all three types of 3D surfaces, Table 1.

Equal weight of all 46 genes in the PCA analysis revealed a clear separation (and most variation) between cells grown at different surface architecture compared to material. Thirteen genes were expressed differently in cells from 2D PS and 2D PDMS surfaces. The most interesting observation was an increase in *CD163* and *IL23A*, and a decrease in *FLT3* and *LAMP3* expression in 2D PDMS cells compared to 2D PS. As *CD163* have been described as a monocyte/macrophage marker [6,31] and *FLT3* and *LAMP3* as markers expressed by DCs [32,33], it seems as if the cells cultured on PDMS in contrast to PS had an expression profile similar to the expression profile of macrophages, and that these cells had an increased activation state in the form of inflammatory cytokine expression even without the addition of LPS. Additionally, LPS-stimulated cells cultured on all three types of PDMS surfaces, had a decreased expression of *BATF3* compared to the control surface. *BATF3* is typically expressed by bona fide DC at higher levels (Gael Aurey and Artur Summerfield, unpublished data) and the downregulation on PDMS surfaces is in accordance with the cells having expression profiles similar to what would be expected for macrophages. It is important to emphasize however, that it cannot be concluded based on these studies alone if the cells cultured on PDMS in fact developed into macrophages. For that conclusion to be made, additional functional studies would have to be included.

It was surprising to find expression of *FLT3* in the monocyte-derived cells as a previous study found this gene to be primarily expressed by bona fide DCs originating from bone marrow DC-precursors [32]. An explanation could be that conventional DCs (cDCs) were present in our monocyte population to begin with as these cells also express CD172a (which was used to purify the monocytes), although at a much lower level than monocytes [34]. Changing the surface to PDMS had a negative effect on the *FLT3* expression.

Adding a third dimension to the culture, i.e. 3D PDMS generated cells that in addition to the above mentioned differences in gene expression, also differed from cells on 2D PS by increased expression of *IL6*, *IL8*, *IL10* and *CCR5*, inflammatory and regulatory cytokines and a chemokine receptor reflecting cells with higher activation status. In addition to *LAMP3*, also *CD1a* and *FCGR2b* which is usually expressed by DCs were downregulated. As shown in the PCA plot, 3D PDMS and pyrolysed 3D PDMS surfaces constituted one cluster. In fact there was not much difference in their gene expression profile; only 2 genes were differently expressed compared to control on 3D PDMS but not on the pyrolysed version of the surface for non-stimulated cells. Also the level of expression was similar on the two surfaces. So the carbonisation of PDMS had only minor effect on the gene expression.

Finally the effect of changing only the culture dimension was tested on the 3D PS surface. This surface clustered differently from the other surfaces and resulted primarily in downregulation of genes compared to control (12 genes were downregulated, 5 were upregulated). Cells from this surface were in an activated state based on upregulation of inflammatory and regulatory cytokines/chemokines as was the case for all 3D surfaces. Apart from the downregulation of the two DC markers, *CD1a* and *FCGR2b*, cells on this surface also downregulated the expression of the macrophage marker *CD163* which were upregulated on all the other alternative surfaces. This emphasises that the skewing of cells toward a gene expression profile closer to macrophages than moDCs, is dependent on surface material (PDMS) more than dimension (3D).

The moDCs generated in this study were slightly adherent to plastic. A common way to distinguish moDC from monocyte-derived macrophages (MDM) is the stickiness of the two cell types; MDM tend to stick more to plastic surfaces than moDCs. It is likely that 3D cultures only are favourable for cells that can exploit the extra dimension by binding to the scaffold. Since we observed both a change in gene- and surface marker expression in cells cultured in 3D compared to 2D, this suggest that the cells do interact- and responds to the extra dimension in the cultures. The stickiness of the cells after culture was not investigated though and it was chosen to include all cells that could be harvested using a mix of ice-cold PBS containing EDTA.

The harvesting process is a concern when working with cells cultured in 3D scaffolds. It is obviously more complicated collecting cells cultured in a 3D setup compared to 2D. There is a risk of losing cells that are stuck in the scaffold and thus basing the further analysis on a fraction of the original cell pool. In this study, an equal number of cells were originally pipetted in each culture well. The cells were not counted after harvesting, but the quantity of total RNA after purification was measured, and this yield can be used as an indirect measure of initial cell quantity. All culture surfaces resulted in a relatively high total RNA yield, except for the 3D PS surface. This scaffold is a commercial available 200 μm thick porous PS membrane made using emulsion templating [35] and designed for 3D culture [36]. The scaffold has a porosity of 90% and the pore size is approximately 40μm. This surface is more compact than the 3D PDMS and pyrolysed 3D PDMS scaffolds which have pore sizes of 300–600μm. We may only speculate if cells were left trapped within the pores of the 3D PS scaffold due to their relative compact architecture compared to 3D PDMS and pyrolysed 3D PDMS. The low RNA yield from 3D PS could also have to do with the material as a higher RNA yield from cells cultured on 2D PDMS compared to 2D PS was obtained. In a study on human breast cancer cells Zhang and colleagues reports a poor cell attachment on plasma oxygen-treated 2D PDMS compared to 2D PS surfaces [37]. Thus the decreased RNA yield after retrieval of cells from 3D PS is probably due to a combination of cells sticking better to the polystyrene and being “trapped” in the relatively small pores of the scaffold. In any case, it seems as if the 3D PS scaffold is preferable for assays that do not require retrieval of cells after culture.

In conclusion, the effect of changing the culture setup when generating porcine moDC was tested and it was found that both material (PDMS) and architecture (3D) changed the gene expression of the cells. Even though one could wish for more biological replicates, multivariate analysis (PCA) suggest that architecture (2D vs. 3D) rather than material (PS vs. PDMS) results in differentially expression of genes involved in pattern recognition and inflammation. Even though we cannot conclude that cells cultured on PDMS were in fact macrophages based on their gene expression pattern alone, we have showed that these cells differed from cells cultured conventionally and that this difference in gene expression was dependent on surface material (PDMS) more than dimension (3D). These findings provide evidence of the necessity of detailed reporting of the culture conditions used when presenting gene expression results as it was found to influence the expression, which make comparisons difficult. The findings also highlight the challenges for future studies combining the analysis or generation of cells having specific phenotypes with for example microfluidic cell culture-devices fabricated using other materials than PS.

## Supporting Information

S1 Table List of primers used in this study. (DOCX)
