# Supplementary material for: Investigating the Role of Surface Materials and Three Dimensional Architecture on In Vitro Differentiation of Porcine Monocyte-Derived Dendritic Cells
Source: PLoS One. 2016 Jun 30;11(6):e0158503. doi: 10.1371/journal.pone.0158503 (PMC4928952; doi:10.1371/journal.pone.0158503)
Supplement: S1 Table — (DOCX) [file pone.0158503.s001.docx]

| **S1 Table: List of primers used in this study.**  87 primers are listed (9 primers were excluded as they did not work). | | | | | | |
| --- | --- | --- | --- | --- | --- | --- |
| **Gene symbol** |  | **Gene name** |  | **Sequence** |  | **Amplicon length** |
| ACTB | | β-actin | | F: CTACGTCGCCCTGGACTTC  R: GCAGCTCGTAGCTCTTCTCC | | 76 |
| B2M | | β-2-microglobulin | | F: TGAAGCACGTGACTCTCGAT  R: CTCTGTGATGCCGGTTAGTG | | 70 |
| GAPDH | | Glyceraldehyde-3-phosphate dehydrogenase | | F: ACCCAGAAGACTGTGGATGG  R: AAGCAGGGATGATGTTCTGG | | 79 |
| HPRTI | | Hypoxanthine phosphoribosyl-transferase I | | F: ACACTGGCAAAACAATGCAA  R: TGCAACCTTGACCATCTTTG | | 71 |
| RPL13A | | Ribosomal protein L13a | | F: ATTGTGGCCAAGCAGGTACT  R: AATTGCCAGAAATGTTGATGC | | 76 |
| PPIA | | peptidylprolyl isomerase A  (cyclophilin A) | | F: CAAGACTGAGTGGTTGGATGG  R: TGTCCACAGTCAGCAATGGT | | 138 |
| TBP | | TATA box binding protein | | F: ACGTTCGGTTTAGGTTGCAG  R: CAGGAACGCTCTGGAGTTCT | | 96 |
| YWHAE | | Tyrosine 3-monooxygenase/  tryptophan 5-monooxygenase | | F: GCTGCTGGTGATGATAAGAAGG  R: AGTTAAGGGCCAGACCCAAT | | 124 |
| IL1B | | Interleukin 1b | | F: CCAAAGAGGGACATGGAGAA  R: GGGCTTTTGTTCTGCTTGAG | | 123 |
| IL6 | | Interleukin 6 | | F: TGGGTTCAATCAGGAGACCT  R: CAGCCTCGACATTTCCCTTA | | 116 |
| IL8 | | Interleukin 8 | | F: GAAGAGAACTGAGAAGCAACAACA  R: TTGTGTTGGCATCTTTACTGAGA | | 99 |
| IL10 | | Interleukin 10 | | F: TACAACAGGGGCTTGCTCTT  R: GCCAGGAAGATCAGGCAATA | | 110 |
| IL12A | | Interleukin 12 p35 | | F: GAACTAGCCACGAATGAGAGTTG  R: ACTGCTAAGGCACAGGGTTG | | 114 |
| IL12B | | Interleukin 12 p40 | | F: GACCAGAAAGAGCCCAAAAAC  R: AGGTGAAACGTCCGGAGTAA | | 70 |
| IL23A | | interleukin 23 | | F: GCTGTGATCCTCAGGGACTC  R: TAGAGAAGGCTCCCCTGTGA | | 119 |
| TLR2 | | Toll like receptor 2 | | F: CGGAGGTTGCATATTCCACAG  R: TGTGAAAGGGAACAGGGAAC | | 128 |
| TLR3 | | Toll like receptor 3 / toll-like receptor 3 variant 1 (TLR3) | | F: ACATCTACTGAAAGATCCATTGTGC  R: TCTTCGCAAACAGAGTGCAT | | 148 |
| TLR4(a) | | Toll like receptor 4 | | F: TTTCCACAAAAGTCGGAAGG  R: CAACTTCTGCAGGACGATGA | | 145 |
| TLR4(b) | | Toll like receptor 4 | | F: TGGTGTCCCAGCACTTCATA  R: CAACTTCTGCAGGACGATGA | | 116 |
| TLR7 | | Toll like receptor 7 | | F: AGAAGCCCCTTCAGAAGTCC  R: GGTGAGCCTGTGGATTTGTT | | 93 |
| TLR8 | | Toll like receptor 8 | | F: GCAAAGACCACCACCAACTT  R: ATCCGTCAGTCTGGGAATTG | | 129 |
| TLR9 | | Toll like receptor 9 | | F: CCTGTTCTATGATGCCTTCGTG  R: GGTACCCAGTCTCGCTCCTC | | 144 |
| IFNA | | Interferon alpha 1 | | F: ATCGTCAGGGCAGAAGTCAT  R: CCAGGTGTCTGTCACTCCTTC | | 86 |
| IFNB | | Interferon beta | | F: AGCACTGGCTGGAATGAAAC  R: TCCAGGATTGTCTCCAGGTC | | 83 |
| IFNG | | Interferon gamma | | F: CCATTCAAAGGAGCATGGAT  R: TTCAGTTTCCCAGAGCTACCA | | 76 |
| TNF | | Tumor Necrosis Factor alpha | | F: CCCCCAGAAGGAAGAGTTTC  R: CGGGCTTATCTGAGGTTTGA | | 92 |
| SLA-DRB1(a) | | Major Histocompatibility Complex, Class II, DR Beta 1 | | F: TGACGGTGTATCCTGCAAAG  R: GTAGAACCCGGTCACAGAGC | | 74 |
| SLA-DRB1(b) | | Major Histocompatibility Complex, Class II, DR Beta 1 | | F: GAGGTCTACAGCTGCCGAGT  R: ATCTTGCCCTGAGCAGATTC | | 92 |
| CD86(a) | | CD86 | | F: CATCGTCTGTGTCCTGCAAC  R: CACAGGTGGCTTTGCATCTA | | 82 |
| CD86(b) | | CD86 | | F: GAACAGGAAGGCGAGTGAAC  R: ATCACACTGGGCATCATCAG | | 73 |
| CD40 | | CD40 | | F: TGAGAGCCCTGGTGGTTATC  R: GCTCCTTGGTCACCTTTCTG | | 90 |
| CCR7(a) | | Chemokine (C-C Motif) Receptor 7 | | F: TCCACGTCTGCAAACTCATC  R: GTCGATGCTGATGCAGAGAA | | 83 |
| CCR7(b) | | Chemokine (C-C Motif) Receptor 7 | | F: GCCATGAGCTTCTGCTACCT  R: CCTTGTTGCGCTCGAAGT | | 70 |
| TRAF6(a) | | TNF Receptor-Associated Factor 6,  E3 Ubiquitin Protein Ligase | | F: CTGCCATGAAAAGATGCAGA  R: GCGACTGGGTATTCTCTTGC | | 62 |
| TRAF6(b) | | TNF Receptor-Associated Factor 6,  E3 Ubiquitin Protein Ligase | | F: CCAGTTCCATGCACATTCAG  R: GCGACTGGGTATTCTCTTGC | | 91 |
| CLEC4A(a) | | C-Type Lectin Domain Family 4,  Member A | | F: ATGGGGTTGGAATGATGTTC  R: TCATGGAGAATGTTCCACTCAT | | 91 |
| IRF1 | | interferon regulatory factor 1 | | F: TGAAGCTGCAACAGATGAGG  R: CTTCCCATCCACGTTTGTCT | | 100 |
| CCR5(a) | | Chemokine (C-C Motif) Receptor 5 | | F: GCCGCAATGAGAAGAAGAAG  R: AGGGAGCCCAGAAGAGAAAG | | 81 |
| CCR5(b) | | Chemokine (C-C Motif) Receptor 5 | | F: CAACTTGCTGGTTGTCCTCA  R: GCCAGGTTGAGCAGGTAGAT | | 78 |
| CXCR4(a) | | Chemokine (C-X-C Motif) Receptor 4 | | F: ACGGGTTCCGTATATTCACTTC  R: GGAAACAGGGTTCCTTTATGG | | 87 |
| CXCR4(b) | | Chemokine (C-X-C Motif) Receptor 4 | | F: CTGCTGGCTGCCATACTACA  R: TCAAACTCACACCCTTGCTG | | 81 |
| IRF8(a) | | Interferon Regulatory Factor 8 | | F: TGGGAGAACGACCAGAAGAG  R: CCAGGCCTTGAAGATGGAG | | 99 |
| IRF8(b) | | Interferon Regulatory Factor 8 | | F: CCGGATTTTGAGGAAGTGAC  R: CTCTTCCTCGGGGACAATG | | 84 |
| FLT3(a) | | Fms-Related Tyrosine Kinase 3 | | F: ATGGATCAGCCATTTTACGC  R: CGTTTCCTGGAGTCAAAAGC | | 77 |
| FLT3(b) | | Fms-Related Tyrosine Kinase 3 | | F: GCTGGAGGAGGAAGAGGACT  R: TCCCTTTGGCCACTTGATAG | | 77 |
| SIGLEC5(b) | | Sialic Acid Binding Ig-Like Lectin 5 | | F: ACGCCTCGATCAAGGTCAC  R: AGCTGAGTCTGAGGCTGGAG | | 96 |
| CCL2 | | Chemokine (C-C Motif) Ligand 2 | | F: GCAAGTGTCCTAAAGAAGCAGTG  R: TCCAGGTGGCTTATGGAGTC | | 103 |
| CD209 | | CD209 | | F: CGGAGCAGAAATTCCTGAAG  R: CATTGCCAGGAACCTTCATT | | 94 |
| ITGA4(a) | | Integrin, Alpha 4 (Antigen CD49D,  Alpha 4 Subunit Of VLA-4 Receptor) | | F: TCCAGAGCCAAATCCAAAAG  R: GCGTTTGGGTCTTTGATGAT | | 94 |
| CCR1(a) | | Chemokine (C-C Motif) Receptor 1 | | F: CTGGCCATTTCTGACCTGAT  R: ACACATGTGATCGCCAAAAA | | 93 |
| CCR1(b) | | Chemokine (C-C Motif) Receptor 1 | | F: TCCAAGAATCCCTGTTCACC  R: AGGCGATGACCTCTGTCACT | | 81 |
| IRF5(a) | | Interferon Regulatory Factor 5 | | F: TCTTCAGCCTGGAGCATTTT  R: CTCCCCAAAGCAGAAGAAGA | | 98 |
| IRF5(b) | | Interferon Regulatory Factor 5 | | F: AACCCCGAGAGAAGAAGCTC  R: CAAGAAAGCTCCCCTGAGAA | | 88 |
| NFKB1 | | Nuclear Factor Of Kappa Light Polypeptide Gene Enhancer In B-Cells 1 | | F: CTCGCACAAGGAGACATGAA  R: GGGTAGCCCAGTTTTTGTCA | | 97 |
| BATF3(a) | | Basic Leucine Zipper Transcription Factor, ATF-Like 3 | | F: GTTCTGCAGAGGAGCGTTTC  R: TCTCTCCTTCGGACCTTCCT | | 86 |
| BATF3(b) | | Basic Leucine Zipper Transcription Factor, ATF-Like 3 | | F: AGGAAGGTCCGAAGGAGAGA  R: TTTGTCAGCCTTCTGGGTTT | | 78 |
| ID2(a) | | Inhibitor Of DNA Binding 2, Dominant Negative Helix-Loop-Helix Protein | | F: GGACATCAGCATCCTGTCCT  R: AGAGCGCTTTGCTGTCACTT | | 74 |
| ID2(b) | | Inhibitor Of DNA Binding 2, Dominant Negative Helix-Loop-Helix Protein | | F: CCAGTGAGGTCCGTTAGGAA  R: GTTGTACAGCAGGCTCATCG | | 99 |
| BCL11A(a) | | B-Cell CLL/Lymphoma 11A  (Zinc Finger Protein) | | F: ATGCGAGCTGTGCAACTATG  R: GTAAACGTCCTTCCCCACCT | | 88 |
| BCL11A(b) | | B-Cell CLL/Lymphoma 11A (Zinc Finger Protein) | | F: GAATTCTCGCCCGAACCTC  R: ACACGTGAGGAGGTCATGGT | | 99 |
| BCL6(a) | | B-Cell CLL/Lymphoma 6 | | F: ATCGTCAACAGGTCCCTGAC  R: GGGTGCAGGTAGAGTGGAGA | | 71 |
| BCL6(b) | | B-Cell CLL/Lymphoma 6 | | F: CAAACCTGAAAACCCACACC  R: ATGGGCCACCTGTACAAATC | | 89 |
| TCF4(a) | | Transcription Factor 4 | | F: GAGTGATAAGCCCCAGACCA  R: TTCCTTTCTCGGACTTGCTG | | 87 |
| TCF4(b) | | Transcription Factor 4 | | F: TCCAGTCTTCCTCCGATGTC  R: GGCAGGAGGTGTACAGGAAG | | 78 |
| MYD88 | | myeloid differentiation primary response protein 88 | | F: CCAGACTAAGTTTGCACTCAGC  R: AGGATGCTGGGGAACTCTTT | | 99 |
| LY96 | | Lymphocyte Antigen 96 (MD2) | | F: CAGTAAAGGTTGAGCCCTGTG  R: TTTGCGCATTGGTAAAGTCA | | 140 |
| CLEC1A(a) | | C-Type Lectin Domain Family 1,  Member A | | F: CCAACACTCAGCAAGACAGC  R. TTCCTGTTCTGGGTTTGGAG | | 91 |
| CLEC2D(a) | | C-Type Lectin Domain Family 2,  Member D | | F: GTCACCACATCACGTTTGGA  R: GGCACATTCTCCAACTCCTC | | 82 |
| CLEC2D(b) | | C-Type Lectin Domain Family 2,  Member D | | F: CGCAAAGAACTGGACAGTCA  R: TTCAGCTCCTCTTCGGTTTC | | 84 |
| CXCL10 | | Chemokine (C-X-C Motif) Ligand 10 | | F: CCCACATGTTGAGATCATTGC  R: GCTTCTCTCTGTGTTCGAGGA | | 141 |
| CXCL2 | | Chemokine (C-X-C Motif) Ligand 2 | | F: GAAGATGCTAAACAAGAGCAGTG  R: AGCCAAATGCATGAAACACA | | 147 |
| CD163(a) | | CD163 | | F: CACATGTGCCAACAAAATAAGAC  R: CACCACCTGAGCATCTTCAA | | 130 |
| CD163(b) | | CD163 | | F: GGAAGTGAGCAGGTCTGGAG  R: ACTCTGGTTCCCTGAGCAGA | | 141 |
| IRF3 | | Interferon Regulatory Factor 3 | | F: GTCAAGAGGCTGGTGATGGTC  R: CTGTTGGAAATGTGCAGGTC | | 119 |
| FCGR1A(a) | | Fc Fragment Of IgG, High Affinity Ia, Receptor (CD64) | | F: GGCAGTGATCACCTTGCAG  R: ATGGGGTCCCTCACATTGTA | | 79 |
| FCGR1A(b) | | Fc Fragment Of IgG, High Affinity Ia, Receptor (CD64) | | F: GCCACAGAAGATGGAAAGGT  R: CGACATGAAACCAGACAGGA | | 94 |
| FCGR2B(a) | | Fc Fragment Of IgG, Low Affinity IIb, Receptor (CD32) | | F: AGGAGTGACTGGGCTGATTG  R: AACAGGAGCCAGGAATAGCA | | 84 |
| FCGR2B(b) | | Fc Fragment Of IgG, Low Affinity IIb, Receptor (CD32) | | F: ACCACCCAGTGGTTCCATAA  R: TCTCCTGGCCTTAAAGCTGA | | 75 |
| FCGR3B(a) | | Fc Fragment Of IgG, Low Affinity IIIb, Receptor (CD16b) | | F: CCGAAGTCTGTGGTGATCCT  R: CCTGGCACTTCAGAGTCACA | | 76 |
| FCGR3B(b) | | Fc Fragment Of IgG, Low Affinity IIIb, Receptor (CD16b) | | F: ACCACATTCCAAATGCAACA  R: ACTTTCACAGCCTCCGAAGA | | 94 |
| CD1A(a) | | CD1a | | F: TAGGAGGCCAGGACATCATC  R: GGCACAATCACTGCCAATAA | | 76 |
| CD1A(b) | | CD1a | | F: CACGTCTCTGGCTTCTACCC  R: GATGTCACCTTGCTGAGTGC | | 87 |
| LAMP3(a) | | Lysosomal-Associated Membrane Protein 3 | | F: TTTGGAAATGTGGACGAGTG  R: ACAATCAAACCCACAGCACA | | 95 |
| LAMP3(b) | | Lysosomal-Associated Membrane Protein 3 | | F: GCACTCCTTCAAGTGCGTAA  R: CCTGAAGCTGGACGTTCATT | | 83 |
| XCR1(a) | | Chemokine (C Motif) Receptor 1 | | F: CTGCTGAAACTTGGGGTCAT  R: CAGTGGGAGAAGGCAATGTT | | 92 |
| XCR1(b) | | Chemokine (C Motif) Receptor 1 | | F: CTGGATTACGCCTTGCTCAT  R: AGACATAGAGCACCGGGTTG | | 73 |
| CD101(a) | | CD101 | | F: ATTGAAACTCAGGCCCACAG  R: CCTGGCTGTGTTCTGTAGCA | | 80 |
